# Competition and quality of care under regulated fees: evidence from Ghana

**DOI:** 10.1186/s13561-022-00406-7

**Published:** 2022-11-10

**Authors:** Adolf Kwadzo Dzampe, Shingo Takahashi

**Affiliations:** 1 Japan Development Service (JDS) PhD Fellow, Graduate School for International Development and Cooperation, Hiroshima University, 1-5-1 Kagamiyama, Higashi-Hiroshima, Hiroshima, 739-8529 Japan; 2 Claims Processing Centre, National Health Insurance Authority, PMB Ministries, Accra, Ghana; 3 Associate Professor, Graduate School for International Development and Cooperation, Hiroshima University, 1-5-1 Kagamiyama, Higashi-Hiroshima, Hiroshima, 739-8529 Japan

**Keywords:** Competition, Physician-induced demand, Doctor-to-population ratio, Quality of care, Hypertension, Ambulatory care sensitive hospitalization, Ghana

## Abstract

**Background:**

How competition affects the quality of care is still not well understood empirically because of limited and mixed results. This study examined whether competition leads to higher or lower quality health outcomes in Ghana.

**Methods:**

We used administrative claims data of hypertension patients for 2017 – 2019 (36 months), and an instrumental variable method to examine the effect of competition, measured as an increase in district doctor-to-population ratio on hospital-level ambulatory care sensitive condition hospitalization and in-hospital death rates.

**Results:**

Overall, we found that an increase in doctor density improves the quality of care for hypertension patients in Ghana. That is, when there are more doctors, fewer patients are hospitalized, and the risk of in-hospital deaths decreases. This result is robust to analyses at the individual and district population levels for ambulatory care sensitive hospitalizations rate.

**Conclusions:**

Our findings suggest that in the presence of physician-induced demand, competition can lead to improvement in the quality of care, possibly through improved access to healthcare and increased physician time and contact per patient. Future health policies need to consider possible welfare benefits of induced medical services and training more doctors.

## Introduction

Due to information asymmetry, the health economics literature has primarily emphasized on the possibility that faced with competition, physicians may induce demand for their financial self-interest, which may lead to escalation in costs.^1^ One important issue that has received less attention is how this asymmetric information affects the quality of health care. As noted by Labelle et al. [1], a more important issue is the quality of the “induced” services. A limited but growing number of empirical studies have sought to provide insight into whether competition leads to higher or lower quality health outcomes, but results have been mixed [2–6].

Existing theoretical literature on quality competition in healthcare markets with regulated prices predicts that competition increases quality of care (when prices are above marginal cost) [7]. For example, an increase in physician density (increased competition) should improve access to healthcare and increase the physician time and contacts per patient. The increased time and contacts should enable physician to follow medical guidelines and the quality per patient should increase. Also, increased physician density (which decreases the number of patients per physician) should increase competition among physicians for patients. If patients value quality and fees are fixed, physician compete by increasing quality [8].

However, how competition affects quality of care is still not well understood empirically [9]. There are several reasons why increase competition may lead to lower quality of care in Ghana. In the presence of physician-induced demand (PID), increased competition provides greater incentives for physicians to provide medical services that may not be beneficial (or even harmful) to patients in their quest to defend income [10]. For example, physicians may commit more patients to hospitalization or cut back on essential inputs such as tests to preserve their incomes as competition increases. In the case of hypertension care in Ghana, for example, past studies show that physicians increase the intensity of treatment when they face increased competition, by prescribing more classes of antihypertensive and other medications [11]. If this is partly motivated by financial self-interest, it could have negative implications for quality of care.

Indeed, there is growing evidence that patients are not satisfied with quality of care they receive from accredited health providers [12–14]. Patients usually perceive health services under the NHIS as “inferior” [12, 15]. As a result, there is wide-spread provider shopping (multiple visits within short period of time) [16, 17]. However, one challenge with many of these studies is that they are surveys which only reflect patients’ perceptions of quality, rather than clinical quality. In addition, patients may not be able to observe all dimensions of quality, especially when studies show that the medical literacy rate in Ghana is quite low [18]. However, to our knowledge, there is no empirical evidence of the relationship between competition and quality of care in Ghana.

Thus, the goal of this paper is to add new evidence from Ghana on whether competition leads to higher or lower quality health outcomes. As prior studies on PID show, an increased competition leads to PID [11, 19–21]. There is evidence from Ghana that, when the doctor/population ratio at the district level increases, physicians react to this increased competition by inducing patients to visit more [11]. Instead, the focus of this study is whether increased competition, captured by an increase in doctor/population ratio, increases or decreases the quality of health outcomes.

To measure health outcomes, we used the risk of in-hospital deaths and ambulatory care sensitive conditions (ACSC) hospitalization for patients with hypertension. We focused on hypertension because it affords doctors a high level of discretion, and deviations from optimal quality are more likely [19]. In Ghana, hypertension is among the leading causes of hospital visits, and prevalence is projected to increase [22]. Under Ghana’s healthcare system, patients are free to see any health provider, and fees are regulated with no cost sharing. Intuitively, increased competition might force physicians to increase the quality of care to attract more patients. We present a formal illustrative model in subsequent section.

Using NHIS administrative claims data for 36 months (2017–2019), we examined the relationship between district-level doctor/population ratio and our outcome measures of quality; namely, the risk of in-hospital deaths and ACSC hospitalization for hypertension patients at the hospital level. Hospitalization for ACSC, also known as avoidable or potentially preventable hospitalization is a common indicator of the effectiveness of primary care [23, 24]. Hypertension and diabetes are examples of chronic ACSC. When properly managed, these conditions do not require hospitalization. Timely access to healthcare, medications, and patient education by physicians is known to reduce hospitalization for these conditions. Thus, increasing hospitalization rates for these conditions are mostly considered indications of poor quality of care [25].

We deal with the potential endogeneity of the doctor/population ratio using an instrumental variable method, in which we use the presence of medical schools in district and metropolitan districts as instruments. Our instruments are meant to capture the ‘attractiveness’ of a location to doctors as a place to live and practice (we provide further justification for our instrument variables in section three). Similar instruments were used in related studies that dealt with endogeneity of doctor-to-population ratio [8, 19, 20, 26]. We improve the robustness of our results by performing analyses at the individual and district population levels. This is because medical services may be beneficial to individual patients, but may not be socially optimal, and vice versa [27].

We found that increased physician density improves the quality of hypertension care in Ghana, and this persists to the population level for ACSC hospitalizations. That is, when there are more doctors, fewer patients are hospitalized, and the risk of in-hospital deaths decreases. Our findings suggest that even though competition may incentivize health providers to act more in their financial self-interest (as suggested by past studies), there is no evidence that such behaviour has negative effect on the quality of hypertension care in Ghana. Rather, access to healthcare, and physician time and contact per patient may have improved. This finding has implications for future health policies. The potential benefits of training more doctors are significant.

The rest of the paper is organized as follows: The next section presents a brief review of the related literature. Section three presents the methods, including an illustrative model which motivates our empirical strategy, empirical implementation strategy, data, variables, and descriptive statistics. Section four presents the results. We have discussion and conclusions in section five.

### Related literature

There is growing literature that examine the effect of competition on quality of care. Mortality is the commonly used outcome. For example, Krakauer et al. [2] used Medicare data from the United States and found that physician supply has negligible effects on mortality, except for areas where physician supply levels are very low. Another study found a negative but insignificant relationship between physician density and total mortality rates [3]. In Germany, Sundmacher and Busse [5] found significantly lower avoidable cancer death rates for some cancers (e.g., colon) as physician supply increased.

Other studies have also examined the relationship between physician density and process quality of care [8, 28, 29]. Jürges and Pohl [8] use the degree of adherence to medical guidelines for the management of risk factors for cardiovascular diseases (CVD) and prevention of falls to construct their process quality of care measure. They use German 2004 Survey of Health, Aging and Retirement in Europe (SHARE) data. Contrary to the prediction of their theoretical model, they find weak and insignificant effect of physician density on quality of care. More recently, Vallejo-Torres and Morris [28] conducted similar study in England where they analysed the relationship between physician supply and thirty-five process measures of quality of care covering thirteen medical conditions. Their results show that physician density has a significant positive effect on quality of care, with effects mostly concentrated in indicators of care related to CVDs and arthritis.

Some previous studies also analysed ACSH rates [2, 25, 30, 31]. For example, Laditka (30) find that low physician areas in the USA have high risk of ACSH while adequate physician supply areas have significantly less risk. Kim et al. [31] finds a negative association between primary care physician density and ACSH rate in Korea. Ricketts et al. [25] examine the relationship between ambulatory care sensitive condition admissions rate and structural access to primary care in North Carolina. They measured structural access by the number of primary care physicians and the presence of subsidized clinics. They find that admission rate was highly corelated with income but not with primary care resources. We find no studies from Africa (and Ghana) that directly examine the relationship between physician density and quality of care.

## Methods

### Model

A theoretical model which motivates our empirical strategy was proposed by Jürges and Pohl [8] The intuition of this illustrative model is that more physicians should improve access to medical services (as travel time reduces) and increase physician time and contacts per patient. Increased time and contact should enable physicians to better follow medical guidelines, and the quality of care per patient should increase. Additionally, increased physician density should lead to increased competition among physicians for patients. If patients value quality and fees are fixed (as in Ghana), physicians compete by increasing their quality.

Formally, the model by Jürges and Pohl [8] is as follows: physicians control only the quality of service. Like Ghana, prices are set by regulators. Hence, the model assumes that physicians take prices as given and set quality to maximize their income. Cost depends on quality and increases at an increasing rate. Benefit depends on true quality (which is observable by patients) and increases at a decreasing rate. The model also assumes two binding constraints: the physician must at least break even for every patient treated. In addition, patients have the option of going to other hospitals or not to receive treatment at all, which gives them some minimum benefit. Therefore, patients must receive benefits that are at least equal to their outside option when they visit a physician. The number of patients that demand services from a particular physician depends on the benefits they provide and doctor/population ratio in the district of practice. The physician density is determined exogenously. Increasing quality increases the number of patients, which increases the income of physicians. On the other hand, an exogenous increase in doctor/population ratio decreases the number of patients per physician, which decreases income. Thus, the optimal quality level as the doctor/population ratio increases must increase. The testable prediction of this model is that quality increases with physician density.

### Empirical strategy

We are interested in testing whether competition, measured as an increased doctor/population ratio, leads to improved quality of care. Therefore, we estimate the following model at the health provider level:

1 $${Quality}_{it} = {\beta }_{0} + {\beta }_{1} ln\left(Doc/{Popl}_{ct}\right) + {X}_{it}II + {C}_{ct}\Gamma + {\delta }_{m} +{\lambda }_{y} + {\alpha }_{r }+ {\varepsilon }_{it,}$$

where *Quality*_*it*_ is the measure of health quality outcomes for provider *i* in month *t*. We used two quality measures; the first is ACSC hospitalization rate, calculated as the number of hospitalizations per 100 hospital visits. The second is in-hospital death rate, calculated as the number of in-hospital deaths per 100 hospitalizations. Our main analysis uses provider-level data (i.e., hospital). The computation of the ACSC hospitalization rate uses all hospital visits for hypertension. However, the in-hospital death rate is computed based on the inpatient visits subsample. $Doc/{Popl}_{ct}$ is the doctor/population ratio at the district level, measured as the number of doctors per 10,000 district population. *X*_*it*_ is a vector of provider-level characteristics such as mean age, gender, type, and ownership of hospital. *C*_*ct*_ is a vector of area and organization characteristics (contextual factors) that can be either time-varying or time-invariant; for example, unemployment rate, proportion of population with no education, per capita income, and hospital beds per capita (see Table 1 for the complete list of variables).

**Table 1:** Summary statistics

Sample Population	Health Provider Level				District Level				Patient Level			
	All Visits		Inpatient		All Visits		Inpatient		All Visits		Inpatient	
Variable	Mean	SD^§^	Mean	SD	Mean	SD	Mean	SD	Mean	SD	Mean	SD
ACSC hospitalization per 100 visits	7.341	(15.835)										
In-hospital deaths per 100 hospitalizations			2.157	(6.993)								
ACSC hospitalization per 1,000 population					0.232	(0.256)						
In-hospital deaths per 1,000 population							0.006	(0.013)				
ACSC hospitalization (= 1 if YES)									0.035	(0.184)		
In-hospital death (= 1 if YES)											0.021	(0.143)
*Individual characteristics*												
Mean age	61.696	(5.892)	62.026	(4.855)	61.980	(5.311)	62.102	(4.653)	62.732	(13.944)	60.797	(16.661)
Female									0.754	(0.430)	0.691	(0.462)
Male									0.246	(0.430)	0.309	(0.462)
Gender (F = 1/M = 2)	1.263	(0.136)	1.256	(0.092)	1.260	(0.131)	1.251	(0.087)				
Average length of stay			3.006	(3.919)			2.962	(3.530)			4.558	(7.417)
*Health Provider characteristics*												
Public hospital	0.398	(0.490)	0.374	(0.484)					0.422	(0.494)	0.416	(0.493)
Mission hospital	0.356	(0.479)	0.383	(0.486)					0.399	(0.490)	0.446	(0.497)
Private hospital	0.246	(0.430)	0.243	(0.429)					0.179	(0.383)	0.138	(0.345)
Primary hospital	0.871	(0.335)	0.905	(0.293)					0.889	(0.314)	0.899	(0.302)
Secondary hospital	0.083	(0.276)	0.061	(0.240)					0.056	(0.230)	0.068	(0.252)
Teaching hospital	0.045	(0.208)	0.034	(0.181)					0.055	(0.227)	0.033	(0.179)
*District Characteristics*												
Doctors per 10,000 population	2.243	(2.668)	2.152	(2.648)	1.517	(2.112)	1.560	(2.120)	2.510	(2.762)	2.362	(2.774)
Proportion aged 65 over	0.042	(0.011)	0.043	(0.012)	0.044	(0.012)	1.557	(2.117)				
Registered Nurses per 10,000	11.601	(10.356)	11.738	(10.443)	10.094	(9.060)	10.373	(9.190)	11.996	(10.649)	12.902	(11.152)
*Region characteristics* ^*¶*^												
Hospital beds per 1,000	0.817	(0.317)	0.831	(0.318)	0.877	(0.321)	0.882	(0.319)	0.861	(0.313)	0.919	(0.297)
Proportion no education	20.377	(17.059)	20.324	(16.676)	21.626	(17.340)	21.381	(16.945)	16.870	(12.422)	20.440	(14.644)
Real per capita Income ('000)	23.146	(20.477)	23.007	(20.608)	19.760	(18.911)	20.436	(19.571)	24.852	(21.334)	21.065	(20.277)
Unemployment rate	8.786	(2.279)	8.676	(2.287)	8.362	(2.235)	8.352	(2.218)	8.538	(2.312)	8.032	(2.181)
Proportion travel time < 30 min	37.016	(9.924)	37.427	(9.836)	37.736	(9.426)	38.164	(9.367)	39.553	(9.374)	39.806	(8.547)
Proportion population insured	41.591	(11.799)	42.004	(11.945)	42.489	(12.194)	42.415	(12.056)	42.146	(12.408)	44.324	(12.944)
*Instruments*												
Metropolitan area status	0.261	(0.439)	0.212	(0.409)	0.100	(0.301)	0.109	(0.312)	0.256	(0.436)	0.181	(0.385)
Presence of medical school	0.215	(0.411)	0.182	(0.386)	0.083	(0.276)	0.090	(0.286)	0.247	(0.432)	0.197	(0.397)
N	2,963		2,486		2,093		1,902		1,681,083		59,253	

Real per capita income inflation adjusted (base year: 2018)

^§^*SD* Standard deviation

^*¶*^ Year 2017 figures imputed for 2018 and 2019 except for proportion population insured which has figures for all year

Andersen [32] refers to these contextual factors as predisposing, enabling, and needing characteristics that are important determinants of health outcomes. For example, we may observe better health outcomes in areas where there are more people insured, unemployment rates are lower, and more medical care resources such as hospital beds and nurses are available. [32, 33] *δ*_*m*_ and *λ*_*y*_ are the month and year fixed effects, respectively. We also control for region-level fixed effects*, α*_*r*_, to counter the concern that doctor density may be correlated with time-invariant region characteristics. For example, doctors in regions with high doctor density may develop certain practice styles, such as providing more amenities or seeking a second opinion [34], which might directly affect the quality of healthcare. Including region fixed effects removes bias due to this type of endogeneity.

Even after controlling for region-level fixed effects, the doctor/population ratio may still be endogenous because of time-varying omitted variables, as pointed out in previous studies. [8, 35]. For example, patients in areas with high doctor density may have greater access to general health knowledge about beneficial lifestyle changes through doctors’ advice, and hence may have a lower risk of in-hospital deaths and ACSC hospitalizations [36, 37]. This introduces a negative bias. On the other hand, physicians may locate in health-resource-deprived areas, where the potential need for health care is high since patients are fully insured, or out of ethical considerations [8, 35]. If patients in such areas are less healthy, they will have higher ACSC hospitalizations and in-hospital deaths, introducing a positive bias.

Therefore, our preferred estimation approach is the two-stage least squares (2SLS) method. Suitable instruments are variables that affect the location decisions of physicians but have no direct effect on our outcome measures. We instrument the doctor/population ratio using the presence of medical schools and the metropolitan status of the district. The argument is that starting physicians tend to live in the district where their medical schools are located after graduation, since they would have forged location-specific capital, so that district mobility tends to be low [38–40]. Additionally, in Ghana, the metropolitan districts have advantages in terms of better amenities (such as schools, access to electricity, and water). This makes it an attractive location for physicians both professionally and as consumers [19, 20].

Our instruments also need to be uncorrelated with omitted factors such as health knowledge and health-resource-deprived areas. One concern with this assumption may be that patients in metropolitan areas may have better health knowledge if people in metropolitan areas have more education. However, our model already controls for region-level average education (proportion with no education). Another concern may be that metropolitan areas and areas with medical schools may have more medical care resources. However, our model additionally controls for medical care resources, such as hospital beds per capita and nurse/population ratio. Yet another concern is that healthy individuals may prefer to live in “attractive” areas. One such mechanism may be that more educated people self-select into metropolitan area, and educated people tend to be healthier [32, 33]. However, we already control for the average educational level in each region. We also control for socio-economic indicators such as unemployment rate and per capita income to further minimize the possibility of the instrumental variables being correlated with the error term. Thus, after controlling for these characteristics, our instruments would capture the “attractiveness” of these locations to doctors as a good place to live and practice. Because our models are over-identified, we can test the exclusion restriction assumption using the Hansen *J* statistic.

### Data, variables and descriptive statistics

The main data are from the National Health Insurance Authority’s administrative claims databases from 2017 to 2019 (36 months). It contains information on the patient’s age, date of treatment, and diagnosis, including the International Classification of Diseases (ICD-10) codes, type of treatment, outcome of treatment (whether the patient died in hospital or discharged), information on service providers, among others. We identified hypertensive disease cases using the following ICD-10 codes: I10—I13, and I15 and in a few cases based on descriptions used instead of the ICD-10 codes (e.g., hypertension, essential hypertension, chronic hypertension, hypertension old—about 4% of data). For analyses at the provider level, the individual data were aggregated.

Data on doctors and nurses were obtained from the Ministry of Health. These data were purposely compiled for this study by the Monitoring and Evaluation Unit. Population data were obtained from the US Census Bureau [41] and Ghana Statistical Services (GSS). Data on socioeconomic characteristics of the region for 2017 are from the *Ghana Living Standard Survey Wave 7 (GLSS7)* published by GSS. We obtained data on the number of hospital beds from *the Health Sector in Ghana (Facts and Figures, 2018*) published by the Ghana Health Service. Data on the location of accredited medical schools were obtained from the Medical and Dental Council of Ghana.

Table 1 presents the summary statistics. Our main results are from data analysed at the provider level, which are shown in columns (1) and (2). Column (1) shows summary statistics using all hospital visits for patients with hypertension. The average ACSC hospitalization per 100 hospital visits was 7.3. The average age of patients with hypertension was 62 years. There are on average 2.2 doctors per 10,000 persons. There are 25% more females than males, and the average length stay in hospitalization is three days. Column (2) shows the summary statistics based on inpatient data only. An average of 2.2 in-hospital deaths out of 100 hospitalizations are recorded every month.

Individual-level and district-level summary statistics are shown in Columns (3) to (6). On average, about 3.5% of all hypertension patients were hospitalized, and of the patients who were hospitalized, about 2.1% resulted in in-hospital deaths. Patients who were hospitalized were, on average, slightly younger (61 years) than all hypertension patients who visited the hospital (63 years). (See Table 1 for summary statistics). We also computed the correlation between the two instrumental variables. The correlation coefficient is statistically significant. However, we believe that each instrument represents separate mechanism through which it generates random variation in the endogenous variable which allows us to isolate the causal effect of medical school and metropolitan areas.

## Results

Table 2 presents the main results of the effect of doctor/population ratio on quality of care analysed at the provider level. Column (1) shows the pooled ordinary least square (OLS) result on the ACSC hospitalization rate, which suggests that there is a negative association between the doctor/population ratio and ACSC hospitalization rate, although it is not statistically significant. As we discussed earlier, the doctor-to-population ratio is likely to be endogenous, even after controlling for time and region fixed effects. Column (2) shows our 2SLS estimation results using the presence of a medical school and the metropolitan status of the district as instruments. We first describe some diagnostic statistics. Regarding the test for the relevance of our instruments, we can reject the null hypothesis that coefficients for our instruments are zero in the first-stage regression, with *F* statistics equal to 27.71. We would like to note that this far exceeds the usual rule-of-thumb criterion that *F* statistics should be greater than 10 for strong instruments [42]. We also fail to reject the over-identifying restrictions with a *p*-value eqaul to 0.21 (please see Hansen’s *J* statistics in Table 2). Overall, the tests give us further confidence in our choice of instruments.

**Table 2:** Health provider level estimates of the effect of doctor-to-population ratio on quality of care

Dependent variables:	(1)	(2)	(3)	(4)
	ACSC hospitalization per 100 visits		In-hospital death per 100 hospitalizations	
	OLS	2SLS	OLS	2SLS
ln(Doctors per 10,000 population)	-1.072	-4.04***	0.028	-1.661***
	(0.791)	(1.26)	(0.36)	(0.524)
ln(Proportion aged 65 and over)	1.319	0.393	1.239	0.546
	(3.077)	(3.146)	(1.623)	(1.815)
ln(Registered nurse per 10,000 population)	-0.563	3.478**	-0.568	1.701**
	(1.923)	(1.745)	(.607)	(.758)
Mean age	-0.579**	-0.6**	-0.116	-0.125
	(0.291)	(0.287)	(0.097)	(0.097)
ln(Hospital beds per 1,000 population)	111.685	114.471	16.395	13.509
	(75.324)	(74.365)	(26.005)	(25.802)
ln(Proportion no education)	34.67	34.272	1.049	-0.931
	(22.21)	(21.799)	(6.556)	(6.423)
ln(Real per capita income)	28.899	29.584	4.536	3.609
	(19.871)	(19.5)	(6.968)	(6.91)
ln(Unemployment rate)	26.89**	24.648**	-1.216	-2.976
	(12.425)	(12.254)	(4.431)	(4.535)
ln(Proportion travel time < 30 min)	46.669	51.06	3.354	2.652
	(42.6)	(42.321)	(11.406)	(11.405)
Mission hospital	-3.169	-2.896	-0.461	0.026
	(2.66)	(2.6)	(.739)	(.708)
Private hospital	-2.798	-3.346	-1.281	-1.576*
	(2.46)	(2.651)	(.789)	(.915)
Gender	12.287***	12.06***	13.921**	14.07**
	(3.575)	(3.509)	(6.911)	(6.78)
Secondary hospital	2.519	3.972	3.251*	3.942**
	(2.724)	(2.901)	(1.855)	(1.856)
Tertiary hospital	-2.951	-1.30	-1.122	-.527
	(3.146)	(2.978)	(2.104)	(2.046)
ln(Proportion population insured)	33.091	29.781	6.967	7.181
	(20.235)	(20.365)	(7.582)	(7.761)
ln(Average length of stay)			-0.3	-0.233
			(0.225)	(0.215)
Hansen *J* statistic (p value)	-	1.60 (0.21)	-	1.85 (0.17)
Weakiv (*F* statistic)	-	27.71	-	32.71
Kleibergen-Paap rk LM (p value)	-	8.09 (0.017)	-	7.07 (0.029)

Standard errors are in parentheses clustered at the district level. Estimates include month, year, and region dummies. *Instruments*: Presence of medical school and metropolitan status of the district. Real per capita income (base year: 2018)

^***^ p < *0.01*

^**^ p < *0.05*

^*^ p < *0.10*

According to the results in Column (2), the estimated coefficient for the doctor/population ratio is statistically significant at the one percent level. This coefficient indicates that if the doctor/population ratio increases by one standard deviation from the mean (i.e., 2.24 to 4.91 per 10,000, or a 119% increase), the number of ACSC hospitalization per 100 visits would decrease by 4.8 (computed as (4.04/100)*119), representing a decrease of about 65%. The finding that an increase in doctor density decreases the ACSC hospitalization rate is consistent with the findings from past studies [30, 31].

Column (3) shows the OLS result for the effect of the doctor-to-population ratio on in-hospital death rate. Like the finding for ACSC hospitalization, this result does not show a statistically significant coefficient for doctor density. However, when we apply 2SLS in Column (4), we again find a negative and statistically significant coefficient of -1.66 for the doctor density. That is, if the doctor/population ratio increases by one standard deviation from the mean (i.e., 2.15 to 4.80 per 10,000, or a 123% increase), the number of in-hospital deaths per 100 hospitalizations would decrease by 2.04, that is, a decrease of about 95%. In addition, we would like to note that our instruments appear to be relevant in the first-stage regression with *F*-statistics equal to 32.71, and that we fail to reject the null hypothesis of the overidentifying restrictions with *p*-value equal to 0.17. Overall, our results support the hypothesis that competition increases the quality of care for patients with hypertension. We found that fewer patients were hospitalized, and the in-hospital death rate reduced as doctor density increased, an indication of improved quality of care [24].

### Robustness check

We estimate two additional sets of models to test the robustness of our results to different units of analysis and to evaluate whether medical services are beneficial at both the individual and population levels. This is because not only is there a conflict between individual health and population health in terms of resource allocation [27], but also because past studies have produced mixed results depending on the unit of analysis [3, 8].

First, we aggregate the data to the district level instead of the hospital level and re-estimate Eq. (1), where the outcome variables are ACSC hospitalization per 1,000 district population and in-hospital deaths per 1,000 district population. Table 3 column (1) shows statistically insignificant negative association between the doctor/population ratio and ACSC hospitalization rate. However, the 2SLS results in column (2) shows statistically significant negative relationship between the doctor/population ratio and ACSC hospitalization rate, consistent with our main result. That is, if doctor/population ratio increases by one standard deviation from the mean (i.e., 1.52 to 3.63 per 10,000, an increase of 139%), ACSC hospitalization per 1,000 population would decrease by 0.23 (or 99%).

**Table 3:** District population level estimates of the effect of doctor-to-population ratio on quality of care

Dependent variables:	(1)	(2)	(3)	(4)
	ACSC hospitalization per 1,000 population		In-hospital deaths per 1,000 population	
	OLS	2SLS	OLS	2SLS
ln(Doctors per 10,000 population)	-0.003	-0.163**	0.0002	-0.004
	(0.027)	(0.069)	(0.001)	(0.003)
ln(Proportion aged 65 and over)	0.113	0.119	-0.003	-0.003
	(0.108)	(0.125)	(0.007)	(0.007)
ln(Registered nurse per 10,000 population)	0.098**	0.313***	0.003	0.008**
	(0.038)	(0.12)	(0.002)	(0.004)
Mean age	0.003*	0.003	0.000	0.000
	(0.002)	(0.002)	(0.000)	(0.000)
ln(Hospital beds per 1,000 population)	3.049**	3.308**	0.022	0.026
	(1.265)	(1.582)	(0.053)	(0.051)
ln(Proportion no education)	1.11**	1.088**	0.004	0.002
	(0.451)	(0.469)	(0.012)	(0.011)
ln(Real per capita income)	0.724**	0.761*	0.002	0.002
	(0.336)	(0.417)	(0.013)	(0.012)
ln(Unemployment rate)	0.697**	0.598*	-0.004	-0.007
	(0.324)	(0.317)	(0.019)	(0.018)
Gender	-0.288***	-0.274***	-0.007*	-0.006
	(0.056)	(0.057)	(0.003)	(0.004)
ln(Proportion travel time < 30 min)	2.516***	2.795***	0.028	0.032
	(0.866)	(0.95)	(0.023)	(0.024)
ln(Proportion population insured)	-0.271	-0.346	0.017	0.016
	(0.378)	(0.48)	(0.016)	(0.016)
ln(Average length of stay)	-	-	0.0002	0.0001
			(0.0007)	(0.0006)
Hansen *J* statistic (*p*-value)	-	0.513 (0.47)	-	2.77 (0.10)
Weakiv (*F* statistic)	-	15.80	-	11.52
Kleibergen-Paap rk LM (*p*-value)	-	5.81 (0.05)	-	5.08 (0.07)

Standard errors are in parentheses clustered at the district level. Estimates include month, year, and region dummies. *Instruments*: presence of medical school and metropolitan status of district

Real per capita income (base year: 2018)

^***^ p < *0.01*

^**^ p < *0.05*

^*^ p < *0.10*

Column (3) shows the results of the effect of doctor/population ratio on in-hospital mortality rate, which suggests a positive but statistically insignificant positive association. The 2SLS results in column (4) shows statistically insignificant negative relationship between the doctor/population ratio and in-hospital mortality rate. Some past studies also found statistically insignificant negative relationship between doctor density and mortality rate [2, 3]. Note that the instruments pass the tests of relevance (*F* statistic = 11.52) and overidentifying restrictions (*p*-value = 0.10).

Finally, we also estimate Eq. (1) at the individual level, where the outcome measures of quality are the probability of ACSC hospitalization (equal to 1 if the patient was hospitalized and 0 otherwise) and the probability of in-hospital death (equal to 1 if the patient died during hospitalization and 0 otherwise). Table 4 column (1) shows a statistically significant negative association between physician density and the probability of ACSH. The 2SLS results from column (2) also show that the probability of ACSC hospitalization decreases with increasing doctor/population ratio. Column (3) shows a negligible and statistically insignificant positive association between the doctor density and the probability of in-hospital deaths. The 2SLS results in column (4) however shows that the probability of in-hospital deaths decreases as doctor density increases. Note that all the statistical tests on the instruments in these models turned out as expected, except for column (4) (see bottom of Table 4). The results of this model should be interpreted with caution.

**Table 4:** Individual level estimates of the effect of doctor-to-population ratio on quality of care

Dependent variable:	(1)	(2)	(3)	(4)
	Probability of ACSC hospitalization		Probability of in-hospital deaths	
	OLS	2SLS	OLS	2SLS
ln(Doctors per 10,000 population)	-0.009***	-0.016***	0.0004	-0.016*
	(0.002)	(0.004)	(0.004)	(0.009)
ln(Registered nurses per 10,000	0.014***	0.023***	-0.005	0.015
Population)	(0.003)	(0.006)	(0.008)	(0.012)
Age	0.0004***	0.0007***	0.0003***	0.0003***
	(0.000)	(0.000)	(0.000)	(0.000)
ln(Hospital beds per 1,000	0.057	0.052	0.103	0.13
population)	(0.118)	(0.125)	(0.131)	(0.13)
ln(Proportion no education)	0.081**	0.072**	0.011	0.000
	(0.031)	(0.033)	(0.038)	(0.037)
ln(Real per capita income)	0.005	0.003	0.025	0.031
	(0.03)	(0.032)	(0.037)	(0.038)
ln(Unemployment rate)	0.063**	0.057**	-0.025	-0.036
	(0.026)	(0.027)	(0.029)	(0.03)
ln(Proportion travel time	0.101	0.093	0.037	0.044
< 30 min)	(0.091)	(0.091)	(0.066)	(0.064)
Mission hospital	0.003	0.006	-0.006	0.000
	(0.005)	(0.005)	(0.007)	(0.006)
Private hospital	-0.005	-0.007	0.002	-0.004
	(0.005)	(0.005)	(0.012)	(0.014)
Male	0.013***	0.013***	0.01***	0.01***
	(0.002)	(0.002)	(0.002)	(0.002)
Secondary hospital	0.014***	.017***	.059**	.065**
	(0.004)	(0.004)	(0.025)	(0.027)
Tertiary hospital	-.01	-0.005	-0.003	0.001
	(0.015)	(0.016)	(0.01)	(0.01)
ln(Proportion population insured)	0.002	0.004	0.024	0.021
	(0.033)	(0.036)	(0.045)	(0.048)
ln(Average length of stay)			0.004	0.004
			(0.003)	(0.003)
Hansen *J* statistic (p-value)	-	0.001 (0.97)	-	5.26 (0.02)
Weakiv (*F* statistic)	-	21.21	-	35.04
Kleibergen-Paap rk LM (p-value)	-	5.07 (0.07)	-	5.23 (0.07)

Standard errors are in parentheses clustered at the district level. Estimates include month, year, and region dummies. *Instruments*: Presence of medical school and metropolitan status of the district

^***^ p < 0.01, ** p < 0.05, * p < *0.10*

Our estimates may seem rather large in magnitude. However, given the situation in Ghana, they are not unreasonable estimates because in areas where doctors are lacking, one doctor sees, on average, over 4,500 patients. As such, a small increase in the number of doctors will reduce the burden of each doctor dramatically. It may be the case that, due to insufficient health care in general, Ghana is still facing increasing returns to scale to number of doctors.

## Discussion and conclusions

We examined the relationship between competition and quality of care in Ghana’s healthcare system where fees are regulated, and patients are fully insured with no cost sharing. We used administrative claims data and an instrumental variable method. We performed analyses at the hospital level, and at the individual and district levels to improve the robustness of our results. Overall, we found that an increase in doctor density improves the quality of care for hypertensive patients in Ghana, which is consistent with previous findings [6, 30, 31]. Our findings suggest that even though competition may incentivize providers to act more in their financial self-interest in Ghana [11], there is no evidence that such behaviour has a negative effect on the quality of hypertension care. On the contrary, we find that competition leads to improved quality of care.

Where we measure quality of care as ACSC hospitalization rate, we find that doctor density has statistically significant positive effects at all levels of analyses. Our results suggest that ACSC hospitalization may be highly sensitive to improvements in primary care and that the availability of doctors could play a significant role in reducing ACSC hospitalization for hypertension care in Ghana. Our finding that increased doctor density leads to improved quality of care is consistent with past studies [30, 31]. On the other hand, when we measure quality of care as the risk of in-hospital deaths, we find mixed results depending on the level of analysis. We find statistically significant positive effect of doctor density on in-hospital death rate at the provider level. We failed to find any reliable effects at the individual and district levels, even though the positive effect remained. Some past studies have also found statistically insignificant and negligible effects [2, 3].

One possible mechanism for the positive effect of doctor density on quality of care is that as physician density increases, the number of patients per physician decreases and doctors are able to spend more time per patient (and better adhere to medical guidelines). More doctors in a district also improves timely access to healthcare, leading to improvement in patient health outcomes. An important implication of our finding is that there is positive return to training more doctors in Ghana. Our findings, together with previous studies from the PID literature [11], suggests that medical services are at levels that the fully informed patient would not have chosen to consume but were found to be clinically beneficial to the patient. It is, however, argued that the “consequences” or health outcomes of induced services are more important than inducement per se [1].

A limitation of this study is that improvements in the quality of hypertension care may not necessarily imply improvement in quality for all diseases when doctor density increases. Generalization of the entire healthcare system is not possible. However, hypertension is one of the leading causes of hospital visits in Ghana and is projected to increase [22]. As such, this study may be relevant to policymakers. Additionally, we used only two measures of quality of care. However, it is possible that patients’ evaluation of quality may be in dimensions other than those evaluated here. For example, patients may evaluate quality based on reception by health staff or the timeliness of services at the hospital. Indeed, studies have noted such divergence between technical and perceived quality of care in Ghana [12]. More comprehensive measures that include patient-oriented quality indicators, such as waiting time and experiences with health staff, could be the focus of future research. However, our quality indicators are widely used measures, especially ACSH, which is a widely accepted indicator of the effectiveness of primary care [23]. Future health policies need to prioritise the training of doctors.

## Data Availability

The data that support the findings of this study are available from NHIA, but restrictions apply to the availability of these data, which were used under license for the current study, and so are not publicly available. Data are however available from the authors upon reasonable request and with permission of NHIA.

## Abbreviations

NHIS: National health insurance scheme
G-DRG: Ghana diagnosis related groups
NHIA: National health insurance authority
ACSC: Ambulatory care sensitive conditions
PID: Physician induced demand
GSS: Ghana statistical service

## Footnotes

1 Doctors may not be perfect agents for their patients [10]. Physicians are partly blamed for escalating health care expenditure because they are believed to unduly influence the use of medical services for their financial self-interest – physician-induced demand [10]. As such, much of the attention of policy makers and health economists have been focused on health service access and cost/expenditure control [1]. For example, in Ghana, the primary concern of government has been the escalating health expenditure that has led to the continuous deficits over the past decade [17].
